# Decreased SLC39A1 (Solute carrier family 39 member 1) expression predicts unfavorable prognosis in patients with early-stage hepatocellular carcinoma

**DOI:** 10.1080/21655979.2021.1987131

**Published:** 2021-10-21

**Authors:** Qinglin Zhang, Jiadong Pan, Fangmei An, He Nie, Qiang Zhan

**Affiliations:** Department of Gastroenterology, Wuxi People’s Hospital Affiliated to Nanjing Medical University, Wuxi, China

**Keywords:** SLC39A1, HCC, immunohistochemistry, prognosis, biomarker

## Abstract

Solute carrier family 39, member 1 (SLC39A1) is a member of the zinc-iron permease family and located to the cell membrane, acting as a zinc uptake transporter. However, the clinical impacts of SLC39A1 in early-stage hepatocellular carcinoma (EHCC) have not been defined. In this research, we compared the differential expression of SLC39A1 in EHCC and normal tissues based on tissue microarray, and the clinical significance of SLC39A1 in EHCC was evaluated as well. Compared with adjacent tissues, SLC39A1 was remarkably decreased in paired EHCC tissues. Besides, decreased SLC39A1 expression was significantly associated with several clinic-pathological features and serum biochemical indicators. Furthermore, Kaplan-Meier analysis exhibited that both overall survival (OS) and relapse-free survival (RFS) of patients with low expression of SLC39A1 were notably poorer than that of patients with high expression. Moreover, Cox regression analyses revealed that low expression of SLC39A1 was an independent prognostic factor for OS in patients with EHCC. Subgroup analysis also revealed beneficiary populations benefiting from the prognostic evaluation using SLC39A1 expression. Collectively, we summarized that downregulated expression of SLC39A1 is a worse prognostic factor for patients with EHCC, which can be used as a promising diagnostic and prognostic biomarker for EHCC.

## Introduction

Hepatocellular carcinoma (HCC) is one of the most fatal tumors in the digestive system worldwide, causing more than 30,000 tumor-related deaths in the United States (US) in 2019 according to statistical data organized by the American Cancer Society (ACS) [1]. Besides, some chronic diseases, such as cirrhosis, diabetes and steatohepatitis, and hepatitis virus infections expose patients to a high risk of hepatocellular carcinoma [2,3]. Although remarkable advances have been achieved in comprehensive treatments for HCC, including surgical operation, chemotherapy, radiotherapy, targeted therapy and immunotherapy, the prognosis of HCC patients remains largely poor due to the delay diagnosis. Patients with early-stage hepatocellular carcinoma (EHCC) tend to have encouraging prognostic, which can be treated curatively by surgical resection, local ablation, and liver transplantation. Thus, exploration of diagnostic and prognostic biomarkers for EHCC is crucial for patients and oncology clinicians. Although an increasing number of researches focusing on diagnostic and prognostic indicators have been conducted [4,5], it is still significant to further identify more efficient potential biomarkers.

Solute carrier family 39, member 1 (SLC39A1) encodes a transmembrane transport protein, a member of the zinc-iron permease family. The subcellular localization of SLC39A1 protein is mainly distributed in the organelle and cell membrane, and its subcellular localization is obviously regulated by Zn^2+^ at the post-translational level [6]. SLC39A1 is widely expressed in mammals’ tissues and cells. However, among various tissues, the prostate contains the highest concentration of Zn^2+^, nearly 10 times more than other tissues. SLC39A1 is the primary transporter in prostate epithelial cells that account for this significant accumulation of Zn^2+^ [7]. It has been found that SLC39A1 act as a significant role in prostate cancer, breast cancer, and colorectal cancer [8–10]. However, the expression and prognostic phenotype of SLC39A1 in human EHCC has not been investigated up to now.

Overall, we assumed that SLC39A1 was dys-regualted in EHCC compared with para-tumor tissues and might act as a potential prognostic biomarker. The present study was designed to define the exact expression level of SLC39A1 in EHCC and adjacent liver tissues by utilizing a tissue microarray slide comprising EHCC specimens. Furthermore, we assessed the associations between SLC39A1 expression and clinical characteristics, serum biochemical indicators, and prognosis of EHCC patients. Besides, we also revealed the prognostic factors of EHCC based on Cox regression analysis and conducted subgroup analyses to determine the prognostic values of SLC39A1 in EHCC patients with different clinical characteristics.

## Materials and methods

### Tissue specimens

The tissue microarray slide (HLivH180Su08) was purchased from Outdo Biotech (Shanghai, China). The slides contained 90 EHCC tissues and paired adjacent tissues, which was diagnosed in clinical stage 1 and 2. All EHCC patients were histopathologically diagnosed by pathologists who selected areas with higher cancer cell density of tumor tissues for hematoxylin-eosin staining. Clinical stages were determined based on American Joint Committee on Cancer (AJCC) 7th classification criteria. Ethical approval for the study of tissue microarray slides was granted by the Clinical Research Ethics Committee, Outdo Biotech (Shanghai, China).

### Immunohistochemistry

Immunohistochemistry (IHC) staining was conducted directly on the tissue microarray slide. The primary antibodies used were as follows: anti-SLC39A1 (1:1500 dilution, Cat. ab105416, Abcam). Antibody staining was visualized with DAB and hematoxylin counterstain (ZSGB-Bio). The percentage of positively stained cells was scored on a scale of 0 to 4, namely, 0: <1%, 1: 1–25%, 2: 25–50%, 3: 50–75% and 4: >75%. The staining intensity was scored from 0 to 3, namely, 0: negative, 1: low, 2: moderate, and 3: high. Semi-quantitative analysis of the IHC staining was performed based on the percentage of positively stained cells and staining density by two pathologists and this analysis method has been previously applied [11]. Immunostained sections were snapshot using a microscope (Olympus Corporation).

### Statistical analysis

Statistical analysis was performed by using SPSS 25.0 and Graphpad prism 6.0. Most of the data were analyzed by Student’s t-test, and bar charts show means ± SDs if not stated. The significance of associations between the SLC39A1 expression and clinic-pathological features were analyzed by χ2 test. The correlations between the SLC39A1 expression and serum biochemical indicators were analyzed by Pearson correlation test. For survival, log-rank test was applied to evaluate the difference between the survival curves, and clinic-pathological factors and SLC39A1 expression were analyzed by univariate and multivariate Cox regression models. For all analyses, a two-tailed P value less than 0.05 was considered statistically significant.

## Results

SLC39A1 is a member of the zinc-iron permease family and acts as a zinc uptake transporter. However, the clinical impacts of SLC39A1 in EHCC have not been defined. The present study was designed to define the exact expression level of SLC39A1 in EHCC and adjacent liver tissues by utilizing a tissue microarray slide comprising EHCC specimens. We also assessed the associations between SLC39A1 expression and clinical characteristics, serum biochemical indicators, and prognosis of EHCC patients.

### SLC39A1 expression was downregulated in EHCC tissues

**Figure 1.**
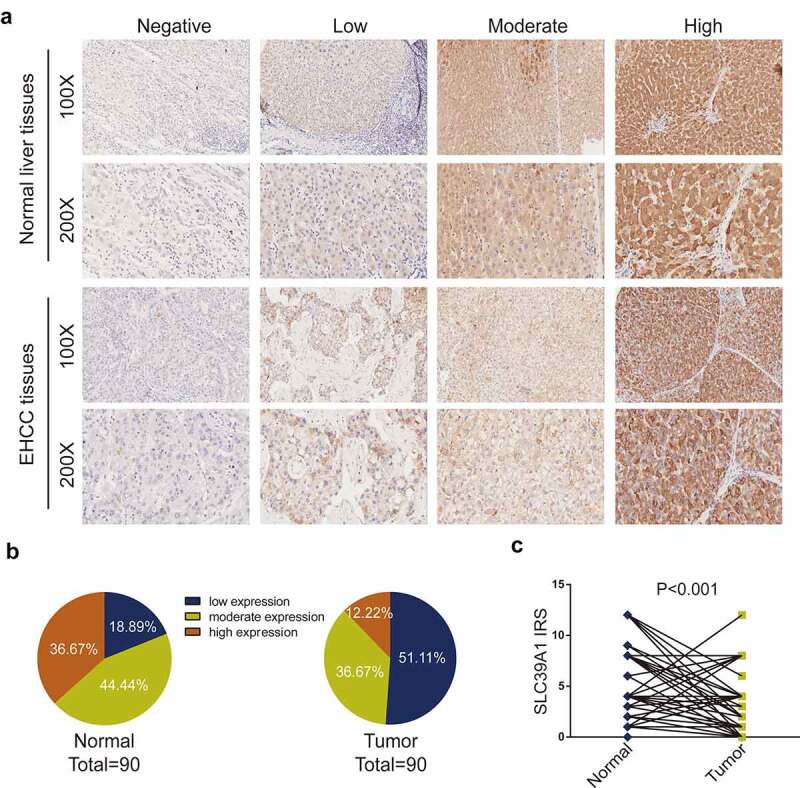
**SLC39A1 expression in EHCC tissues and normal liver tissues**. (a) Staining of SLC39A1 was evaluated by an immunoreactivity score (IRS) by multiplying the intensity score with the score of percentage of positive cells. Representative microphotographs represented low, moderate and high staining intensity in EHCC tissues and normal tissues. Brown, SLC39A1. Blue, hematoxylin. (b) SLC39A1 protein expression intensity proportion of EHCC tissues and paired normal tissues. Low expression: IRS<4; Moderate expression: 4≤ IRS<8; High expression: IRS≥8. (c) The expression of SLC39A1 protein in EHCC tissues and paired normal tissues. A significant decrease of SLC39A1 expression was observed in EHCC tissues compared with paired normal tissues

As described above, several researchers have observed the dysregulated expression of SLC39A1 in prostate cancer, breast cancer, and colorectal cancer [8–10], while the differentiated expression in normal liver and EHCC tissues has not been summarized. We compared SLC39A1 expression based on IHC staining (Figure 1(a)). As shown in Figure 1(a), the immunoreactivity of SLC39A1 was mostly localized to the cytoplasm. After the analysis of 90 EHCC cases, we found that the sections with low and moderate SLC39A1 expression accounted for a majority of tumor tissues (Figure 1(b)). Besides, the IRS of SLC39A1 in EHCC tissues was significantly lower than paired paracancerous tissues (P < 0.001, Figure 1(c)). Overall, all these results revealed that SLC39A1 was decreased in EHCC tissues

### SLC39A1 expression was correlated with clinic-pathological features and serum biochemical indicators

Next, the correlation between clinicopathological features and SLC39A1 expression was evaluated in this patients’ cohort. As shown in Table 1, the expression of SLC39A1 was not associated with gender (P = 0.660), age (P = 1.000), clinical stage (P = 0.208), cirrhosis (P = 0.396) and HBV infection status (P = 0.561). However, differentiated degree (P = 0.012), overall survival (OS) event (P = 0.010) and replapse-free survival (RFS) event (P = 0.032) had tight relationships with decreased SLC39A1. Besides, tumors with poor differentiation expressed low SLC39A1 compared with well-differentiated tumors (Figure S1A-B). In other words, given the advanced tumors tended to express lower SLC39A1, the expression of SLC39A1 was negatively associated with the malignant tendency of the tumor.

**Table 1.** Association between SLC39A1 expression and clinical characteristics in HCC patient

Characteristics	n	SLC39A1^a^		χ2	*P* value
		low	high		
**Gender**					
female	16	7	9	0.193	0.660
male	74	28	46		
**Age**					
≤60	72	28	44	0.000	1.000
>60	18	7	11		
**Differentiated degree**					
well	58	17	41	6.298	**0.012**
poor	32	18	14		
**Clinical stage**					
1	61	21	40	1.586	0.208
2	29	14	15		
**Cirrhosis**					
no	12	6	6	0.719	0.396
yes	78	29	49		
**HBV infection**					
no	13	6	7	0.337	0.561
yes	77	29	48		
**OS event**					
alive	41	10	31	6.661	**0.010**
dead	49	25	24		
**RFS event**					
relapse	30	7	23	4.582	**0.032**
without	60	28	32		

a: low expression in HCC: IRS≤1, high expression in HCC: IRS>1.

**Figure 2.**
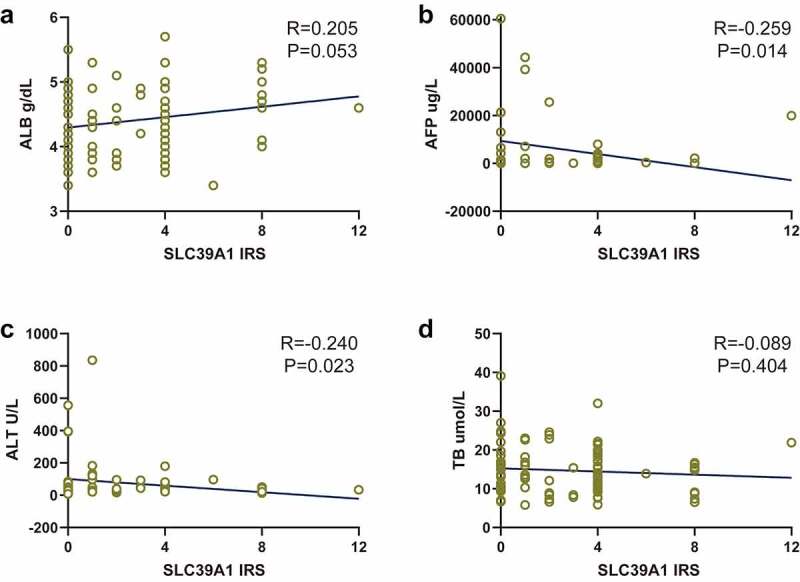
**Correlation between SLC39A1 expression and serum biochemical indicators levels**. (a) SLC39A1 expression had potential positive correlation with ALB level. (b) SLC39A1 expression had negative correlation with AFP level. (c) SLC39A1 expression had potential positive correlation with ALL level. (d) SLC39A1 expression had no obvious correlation with TB level. ALB, albumin; AFP, alpha fetoprotein; ALT, glutamic-pyruvic transaminase; TB, total bilirubin

We next checked the correlations between SLC39A1 expression and serum biochemical indicators levels, which were provided by the original documents. As Figure 2 exhibited, SLC39A1 expression had potential positive correlations with albumin (ALB, P = 0.053, Figure 2(a)), while had negative correlations with alpha fetoprotein (AFP, P = 0.014, Figure 2(b)) and glutamic-pyruvic transaminase (ALT, P = 0.023, Figure 2(c)). There was no obvious correlation between SLC39A1 expression and total bilirubin (TB, P = 0.404, Figure 2(d)).

### Decreased SLC39A1 expression was associated with poor prognosis in EHCC

**Figure 3.**
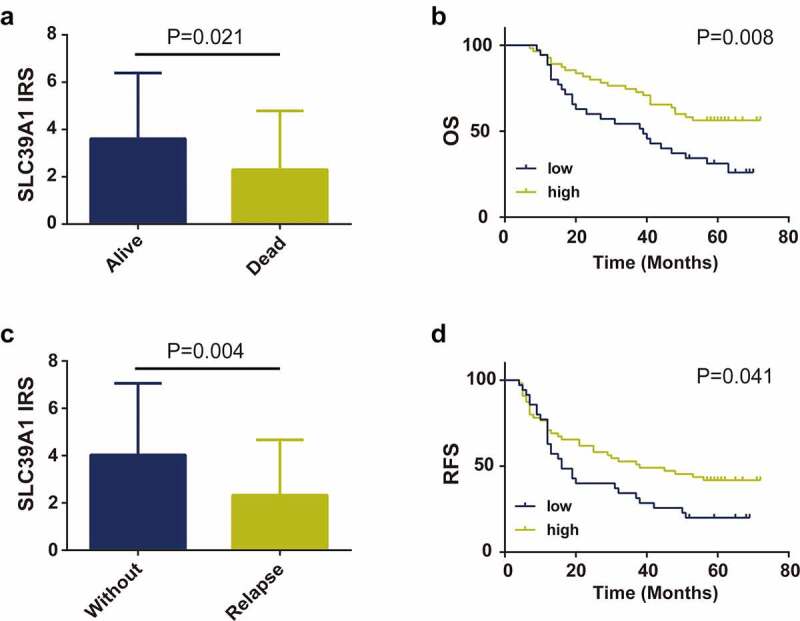
**Prognostic value of SLC39A1 expression in EHCC patients**. (a) Expression of SLC39A1 in alive and dead EHCC patients. (b) Downregulated SLC39A1 expression was associated with poor OS in EHCC patients. (c) Expression of SLC39A1 in patients with our without relapse. (d) Downregulated SLC39A1 expression was associated with poor RFS in EHCC patients

We further definite the prognostic values of SLC39A1 expression in EHCC patients. Firstly, we compared the expression levels of SLC39A1 in alive and dead EHCC patients and the results showed that SLC39A1 expression was significantly downregulated in dead patients (Figure 3a). Similarly, SLC39A1 expression was also decreasing in relapse EHCC patients (Figure 3c). Besides, the Kaplan-Meier analysis was applied to investigate the correlation between SLC39A1 expression and OS and RFS in 90 patients with EHCC. The results showed that both OS and RFS of patients with low SLC39A1 expression were significantly worse than the high expression group (P < 0.001, Figure 3(b,d)). Overall, these results suggested that the decreased SLC39A1 was a poor prognostic factor in EHCC patients.

### Univariate and multivariate Cox regression analyses of prognostic factors for EHCC

To further evaluate the prognostic factors of patients with EHCC, the Cox regression model was utilized for univariate and multivariate survival analyses. We firstly conducted Cox regression analyses to explore the prognostic factors for OS. As shown in Table 2, univariate analysis showed that clinical stages (HR = 1.968, 95%CI: 1.113–3.478,P = 0.020), and SLC39A1 expression (HR = 0.479, 95%CI: 0.273–0.840, P = 0.010) were associated with OS in EHCC patients. In multivariate analysis, we found that only SLC39A1 expression (HR = 0.520, 95%CI: 0.294–0.921, P = 0.025) were independent prognostic factors for EHCC. As for RFS, univariate analysis revealed that clinical stages (HR = 1.747, 95%CI: 1.033–2.955, P = 0.037), and SLC39A1 expression (HR = 0.596, 95%CI: 0.358–0.995, P = 0.048) were also associated with RFS in EHCC patients, but no independent prognostic factor was found in multivariate analysis (Table 3). Overall, these findings revealed the promising value of SLC39A1 serving as an independent prognostic factor for evaluating EHCC patients’ prognosis.

**Table 2.** Univariate and multivariate analysis of survival factors associated with OS in HCC patients

Characteristics	Univariate analysis			Multivariate analysis		
	HR	95%Cl	P value	HR	95%Cl	P value
**Gender**						
female vs. male	1.649	0.740–3.672	0.221			
**Age**						
≤60 vs. >60	1.643	0.870–3.105	0.126			
**Differentiated degree**						
well vs. poor	0.878	0.483–1.596	0.669			
**Clinical stage**						
1 vs. 2	1.968	1.113–3.478	**0.020**	1.777	0.998–3.163	0.051
**Cirrhosis**						
no vs. yes	0.795	0.357–1.775	0.576			
**HBV infection**						
no vs. yes	0.737	0.345–1.572	0.429			
**SLC39A1 expression**						
low vs. high	0.479	0.273–0.840	**0.010**	0.520	0.294–0.921	**0.025**

**Table 3.** Univariate and multivariate analysis of survival factors associated with RFS in HCC patients

Characteristics	Univariate analysis			Multivariate analysis		
	HR	95%Cl	P value	HR	95%Cl	P value
**Gender**						
female vs. male	1.907	0.905–4.021	0.090			
**Age**						
≤60 vs. >60	1.063	0.575–1.966	0.845			
**Differentiated degree**						
well vs. poor	1.386	0.823–2.335	0.220			
**Clinical stage**						
1 vs. 2	1.747	1.033–2.955	**0.037**	1.654	0.994–2.809	0.063
**Cirrhosis**						
no vs. yes	1.006	0.477–2.118	0.998			
**HBV infection**						
no vs. yes	1.036	0.492–2.183	0.926			
**SLC39A1 expression**						
low vs. high	0.596	0.358–0.995	**0.048**	0.630	0.376–1.054	0.079

### Subgroup analysis of the prognostic values of SLC39A1 in EHCC

**Figure 4.**
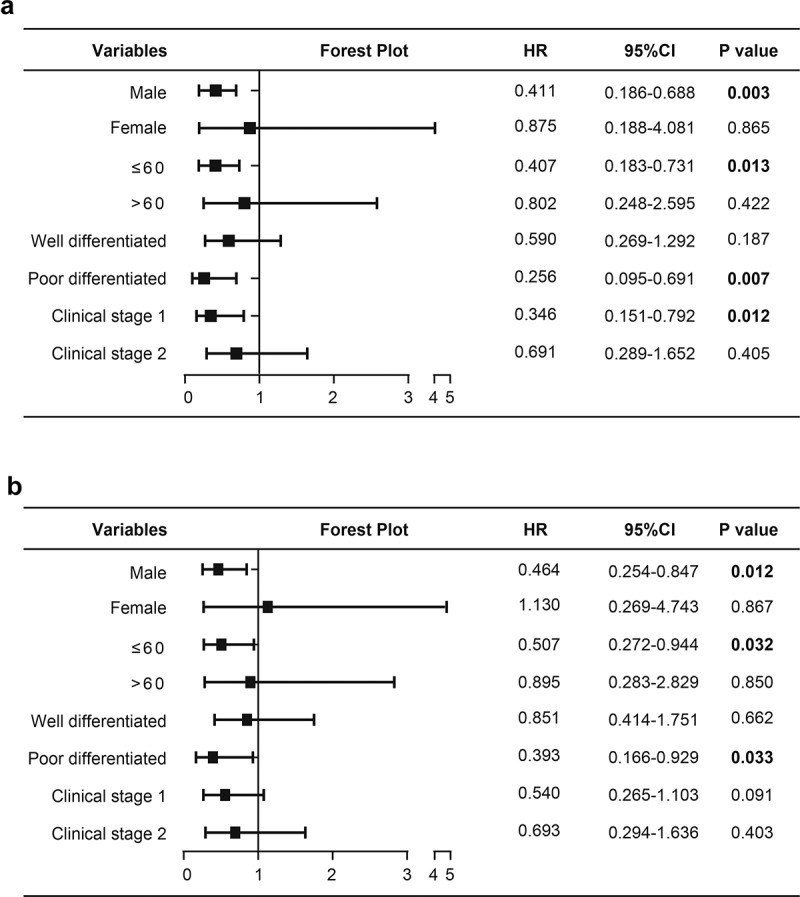
**Subgroup analysis of the prognostic values of SLC39A1 in EHCC patients**. (a) Subgroup analysis of the prognostic values for OS of SLC39A1 in EHCC patients. Downregulated SLC39A1 was correlated with OS in male patients and patients with young age, poor differentiated tumor and clinical stage 1. (b) Subgroup analysis of the prognostic values for RFS of SLC39A1 in EHCC patients. Downregulated SLC39A1 was associated with RFS in male patients and patients with young age and poor differentiated tumor

We next conduct subgroup analysis to find beneficiary populations benefiting from the prognostic evaluation using SLC39A1 expression. The results showed that expression of SLC39A1 was correlated with OS in male patients (P = 0.003) and patients with young age (≤60) (P = 0.013), poorly differentiated tumor (P = 0.007) and clinical stage 1 (P = 0.012) (Figure 4(a)). In addition, subgroup analysis for RFS showed that the expression of SLC39A1 was associated with RFS in male patients (P = 0.012) and patients with young age (P = 0.032) and poorly differentiated tumor (P = 0.033) (Figure 4(b)). Taken together, we defined the prognostic values of SLC39A1 in EHCC patients with different clinical characteristics by subgroup analysis.

## Discussion

As a significant trace element for human body, Zn is involved in the synthesis of various enzymes and acts as an essential role in the proliferation and growth of cells, thus regulating multiple physiological and pathological processes [12,13]. At the cellular level, Zn^2+^ homeostasis is maintained by complex mechanisms including uptake, storage and excretion. In the complex process, the Zn transporter families play critical roles. Currently, two major Zn transporter families have been identified, namely the SLC30 family and the SLC39 family, which control the intra- and extracellular equilibrium of Zn^2+^ [14]. The two major Zn transporter families tend to perform the opposite function in maintaining Zn^2+^ homeostasis. The SLC30 family mainly transports Zn out of the cells, while the SLC39 family mainly contributes to uptake and transport of Zn into the cells. Emerging evidences have shown that the SLC39 family is close to the oncogenesis and development of cancers. Recent studies have suggested that low SLC39A1 [15], SLC39A2 and SLC39A3 [16] expression in prostate cancer, low SLC39A3 [17] and high SLC39A4 [18] expression in pancreatic cancer. However, although an increasing number of researches focused on the roles of the SLC39 family in cancers, the functions of individual members in HCC have not been explored yet.

SLC39A1 is a member of the SLC39 family and is widely expressed in multiple tissues and cells. Although SLC39A1 is expressed widely in mammalian tissues, the tumor-related role of this transporter has been only established in the prostate. A primary study uncovered that tumorigenic prostate epithelial cells (RWPE2) accumulated less intracellular zinc than non-tumorigenic prostate epithelial cells (RWPE1) and this reduction in RWPE2 cells may be due to the decrease in the SLC39A1 protein expression [8]. Franklin *et al*. analyzed the expression pattern of SLC39A1 in several prostate cancer cell lines and speculated that the downregulation is likely own to in situ gene silencing based o the fact that all the malignant cell lines tended to express low SLC39A1 [19]. Moreover, Leslie *et al*. utilized transgenic adenocarcinoma of the mouse prostate model initially characterized the role of SLC39A1/zinc/citrate in prostate cancer [20]. Additional mechanisms of SLC39A1 participating in prostate cancer are also possible. Ectopic expression of SLC39A1 suppressed nuclear factor-κB (NF-κB) in prostate cancer cells in vitro and in vivo and consequently upregulated expression of pro-angiogenic and pro-metastatic cytokines [15,21]. Besides, Ding *et al*. conducted systematical survival analyses of the SLC39 family in gastric cancer and indicated that most members were closely associated with prognosis of gastric cancer patients [22]. However, the function and prognostic role of SLC39A1 in other cancers, especially in HCC have not been observed.

In the current study, we evaluated the expression of SLC39A1 based on tissue microarray of EHCC, and notably downregulated expression of SLC39A1 was found in EHCC tissues compared with paired normal tissues. Besides, decreased SLC39A1 expression was revealed to be associated with tumor differentiated degree, OS event and RFS event. Previous research revealed that high serum levels of AFP and ALT were associated with disease progression and poor prognosis in patients with hepatocellular carcinoma [23,24]. SLC39A1 expression had negative correlations with AFP and ALT as well. Our study also assessed the critical role in the prognosis of EHCC patients. Kaplan-Meier analysis exhibited that the OS and RFS of patients with low expression of SLC39A1 were significantly worse than that of patients with high expression. Besides, both univariate and multivariate Cox regression analyses suggested that low expression of SLC39A1 was an independent prognostic factor for the OS of patients with EHCC. In addition, subgroup analysis revealed that the prognostic value of SLC39A1 expression was more notable in male patients and patients with young ages and poorer differentiated grades.

## Limitations

To best of our knowledge, this research first focused on the dys-expression of SLC39A1 and its prognostic value in EHCC, and the results preliminary suggested the potential tumor-suppressive role in EHCC. Even though our research systematically demonstrated the expression profile and prognostic value of SLC39A1 in EHCC, this research has several limitations as well. The possible mechanisms that SLC39A1 participates in the oncogenesis and progression of EHCC need to be further studied by functional assays.

## Future direction

Recently, Wang et al. revealed that SLC39A1 was upregulated expression in glioma tissues. Besides, SLC39A1 promotes the proliferation of glioma cells, inhibits their apoptosis, and promotes the expression of MMP2 & MMP9. However, in our current research, we found that SLC39A1 might be a potential tumor suppressor in hepatocellular carcinoma. In the future, we will pay more attention to the molecular mechanisms of SLC39A1 in HCC cells behaviors and explore novel therapies for EHCC patients by upregulating SLC39A1 expression.

Moreover, HCC patients with high concentration of Zn^2+^ exhibited better prognosis than these with low concentration [25]. According to Kambe’s report, SLC39A1, SLC39A2 and SLC39A3 play critical roles in zinc homeostasis when zinc is replete, but they play important, non-compensatory roles when this metal is deficient [26]. Thus, the potential mechanisms that SLC39A1-mediated Zn^2+^-dependent oncogenesis in HCC is also an interesting research hotspot.

## Conclusion

To sum up, the present study reported that SLC39A1 was lower expressed in EHCC and decreased expression of SLC39A1 was an independent predictor for poor prognosis of patients with EHCC. As a candidate tumor suppressor, SLC39A1 may be a promising biomarker for early diagnosis, tumor progression, and prognostic assessment by IHC staining.

## Supplementary Material

Supplemental Material

## Data Availability

The data used to support the findings of this study are available from the corresponding author upon request.

## Research highlights

SLC39A1 expression was downregulated in EHCC tissues

SLC39A1 expression was correlated with differentiated degree, OS and RFS event

SLC39A1 expression was negatively correlated with AFP and ALT levels

Decreased SLC39A1 expression was associated with poor prognosis in EHCC
