# Supplementary material for: Decreased SLC39A1 (Solute carrier family 39 member 1) expression predicts unfavorable prognosis in patients with early-stage hepatocellular carcinoma
Source: Bioengineered. 2021 Oct 21;12(1):8147–56. doi: 10.1080/21655979.2021.1987131 (PMC8806984; doi:10.1080/21655979.2021.1987131)
Supplement: Supplemental Material [file KBIE_A_1987131_SM3467.docx]

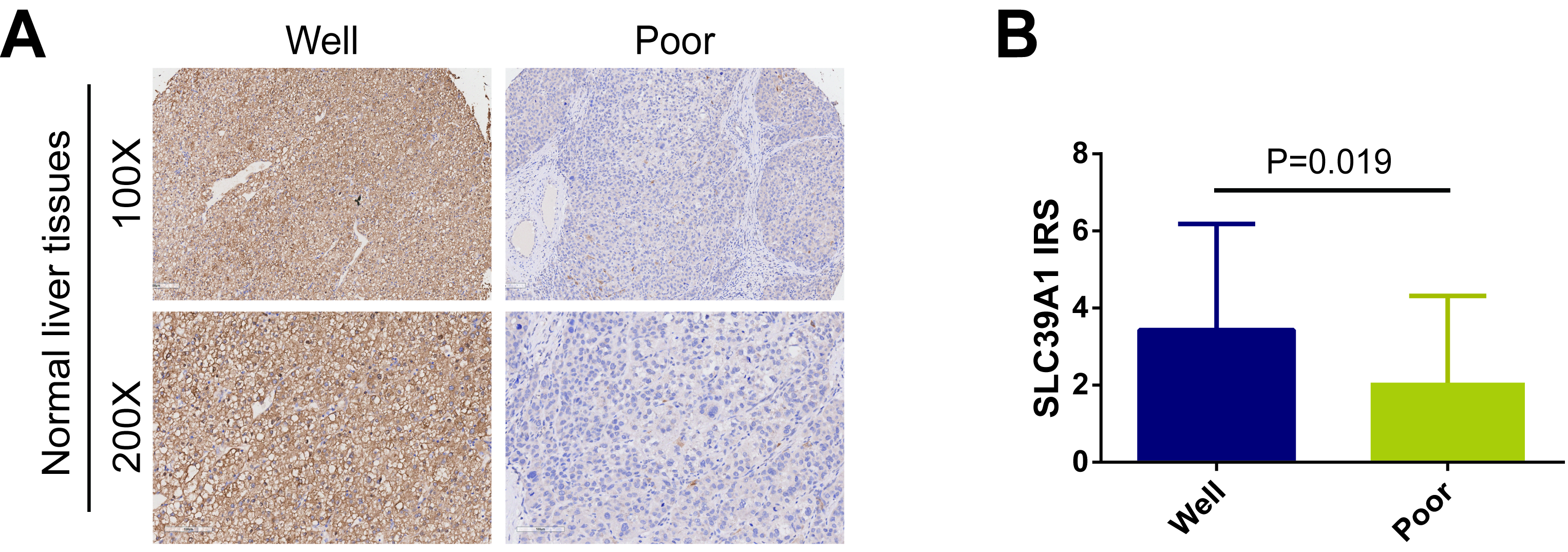


**Figure S1. SLC39A1 expression in EHCC tissues with various differentiated degrees**

(A) Representative microphotographs represented SLC39A1 expression in EHCC with well and poor differentiated degrees (B) Expression of SLC39A1 in these two groups.
